# Increased risk for inflammatory bowel disease in congenital hypothyroidism supports the existence of a shared susceptibility factor

**DOI:** 10.1038/s41598-018-28586-5

**Published:** 2018-07-05

**Authors:** Helmut Grasberger, Mohamed Noureldin, Timothy D. Kao, Jeremy Adler, Joyce M. Lee, Shrinivas Bishu, Mohamad El-Zaatari, John Y. Kao, Akbar K. Waljee

**Affiliations:** 1 Department of Internal Medicine, Division of Gastroenterology, University of Michigan, Ann Arbor, Michigan 48109 USA; 2 Veterans Affairs Center for Clinical Management Research, Ann Arbor, Michigan 48109 USA; 3 Department of Pediatrics and Communicable Diseases, Division of Pediatric Gastroenterology, Michigan Medicine, University of Michigan, Ann Arbor, Michigan 48109 USA; 4 Child Health Evaluation and Research (CHEAR) Center, University of Michigan, Ann Arbor, Michigan 48109 USA

## Abstract

Loss-of-function mutations in dual oxidase (DUOX) 2 are the most common genetic variants found in congenital hypothyroidism (CH), and similar mutations have been recently reported in few very-early-onset inflammatory bowel disease (IBD) patients without CH. If *DUOX2* variants indeed increase susceptibility for IBD, the enrichment of *DUOX2* mutation carriers among CH patients should be reflected in higher risk for developing IBD. Using a database containing health insurance claims data for over 230 million patients in the United States, 42,922 subjects with CH were identified based on strict inclusion criteria using diagnostic codes. For subgroup analysis, CH patients with pharmacy records were stratified as transient or permanent CH based on the absence or presence of levothyroxine treatment, respectively. Patients were matched to an equal-sized, age- and gender-matched non-CH group. Compared to controls, CH patients had a 73% higher overall IBD prevalence (0.52% vs 0.30%; *P* < 0.0001). The CH-associated relative risk was higher for indeterminate or ulcerative colitis than Crohn’s disease. Patients with transient CH had higher odds for IBD (OR 2.39 (95% CI 1.77–3.23) than those with permanent CH (1.69 (95% CI 1.31–2.18). We conclude that patients with CH are at an increased risk of developing IBD. The risk was highest for patients with transient CH, for which partial defects in the DUOX2 system are a particularly common finding.

## Introduction

The follicular cells of the thyroid gland constitutively express a homolog of the phagosomal NADPH oxidase, called dual oxidase 2 (DUOX2) that generates hydrogen peroxide at the apical membrane. By driving thyroid-peroxidase catalyzed iodide organification, this activity is rate limiting for the synthesis of thyroid hormone^1^. In fact, mutations in *DUOX2*, first described in 2002^2^, are now regarded the most common known genetic risk factor in congenital hypothyroidism (CH)^3–7^ found in up to 83% of CH cohorts^8^. The severity of *DUOX2*-associated CH can range from transient neonatal hyperthyrotropinema to permanent profound hypothyroidism.

In addition to being constitutively expressed in thyrocytes, *DUOX2* is robustly induced in the barrier epithelial cells of the gastrointestinal tract as a sentinel response to potential microbial threats. *DUOX2* and its essential heterodimerization partner *DUOXA2* are part of a “*DUOX2* co-expression signature” most responsive to mucosal bacterial dysbiosis^9^ and mice with a defect in the DUOX2 system were found to have increased intestinal bacterial translocation to both commensals and pathogenic bacteria consistent with a role of DUOX2 in supporting immune homeostasis^10^, potentially by suppressing bacterial virulence^11^. By its function and regulation, *DUOX2* is a candidate gene for inflammatory bowel diseases (IBD), chronic inflammatory disorders of the gastrointestinal tract. The most common forms of IBD are ulcerative colitis (UC), which is limited to colonic involvement, and Crohn’s disease (CD), which can involve the entire length of the intestine. The current understanding of IBD pathogenesis is that an altered luminal microbial composition with epithelial dysfunction results in increased bacterial translocation and immune stimulation^12^. Although no traditional pathogens are generally detected during active disease flares, increased virulence of commensal bacterial species, particularly *Escherichia coli*, can enhance their mucosal attachment, invasion, and intracellular persistence, leading to increased pathogen-recognition and mucosal inflammation^12^. Although there is no information from the published literature that any of the previously reported CH patients with *DUOX2* mutations had GI symptoms, anecdotal support for the potential role of DUOX2 in IBD pathogenesis has been recently provided by reports of *DUOX2* loss-of-function mutations in children with IBD but not CH^13–15^. However, *DUOX2* lacks common allelic variants suitable for genome-wide association studies and whether *DUOX2* variants have an impact on IBD risk in the general population is unknown.

Since a high proportion of patients identified in neonatal CH screening programs harbor *DUOX2* variants that may also contribute to the susceptibility for IBD, we hypothesized that the prevalence of IBD would be increased for a CH cohort compared to a matched non-CH cohort. To test this hypothesis, we performed a retrospective cohort analysis using a national payer database in the United States comprising individual-level health care claims data for 230 million individuals.

## Results

### Patient demographics

**Figure 1:**
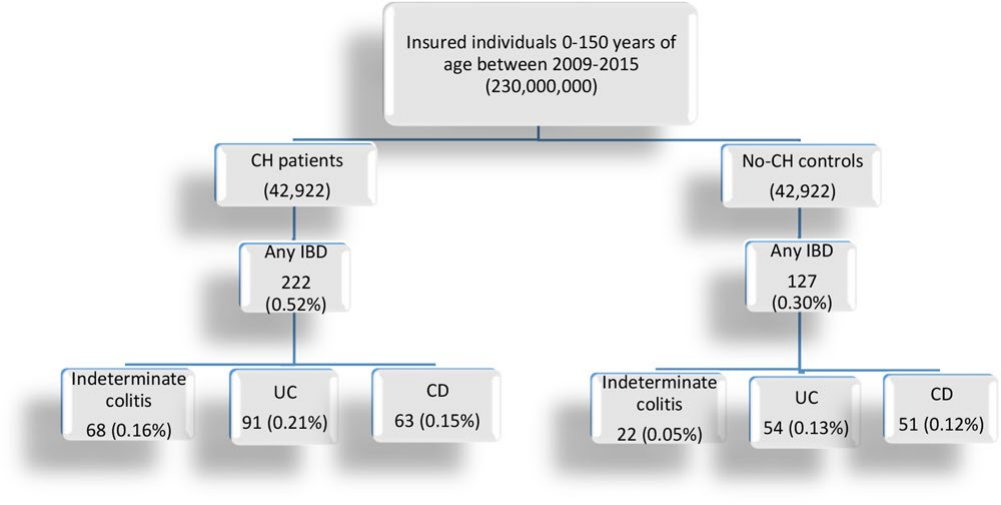
A diagram of the study cohort selection. CH = congenital hypothyroidism, IBD = inflammatory bowel disease, UC = ulcerative colitis, CD = Crohn’s disease.

Using the Truven Health MarketScan databases from 2009–2015, we identified 42,922 patients with CH and 42,922 age-and-gender matched non-CH individuals over the study period (Fig. 1). The mean (±standard deviation) age was 39.1 (25.5) years in each group. The percentage of females was 67.4% in either group. Additional baseline characteristics of patients are provided in Supplemental Table 1.

### Increased prevalence of IBD in subjects with congenital hypothyroidism

To determine whether individuals with CH are at increased risk of developing IBD, we compared the prevalence of IBD in CH vs. non-CH cohorts (n = 42,922). A significantly higher prevalence of patients having any IBD subtype was found in CH vs. non-CH cohorts (p < 0.0001, Table 1). The estimated prevalence of IBD per 100,000 individuals is 520 in CH and 300 in non-CH, indicating a 73% increased risk for developing IBD in CH patients. The higher prevalence was primarily due to increased risk for indeterminate colitis or UC, with only a trend towards more frequent occurrence of CD (Table 1). We found no significant difference in IBD severity based on the rate of hospitalization and IBD flare (data not shown).

**Table 1:** The prevalence of IBD in patients with CH vs. non-CH controls.

	CH N = 42,922	non-CH N = 42,922	p value
CD	63 (0.15%)	51 (0.12%)	0.2607
UC	91 (0.21%)	54 (0.13%)	<0.0021
Indeterminate Colitis	68 (0.16%)	22 (0.05%)	<0.0001
Any IBD	222 (0.52%)	127 (0.30%)	<0.0001

### Sensitivity Analyses

To apply a stricter definition of CH, we applied a 3 ICD code requirement for CH and found similar results of an increased prevalence of any IBD in those with CH compared to those with no CH (0.52% vs. 0.28%; p < 0.0001) and stronger association with indeterminate colitis and UC (Supplemental Table 2).

### Transient CH patients have the highest risk of developing IBD

Children with CH that regain normal thyroid function during early life are classified as transient CH. Whereas permanent CH is caused by a variety of genetic defects in addition to *DUOX2* (e.g., *TPO*, *TG*, *PAX8*) as well as sporadic developmental abnormalities (e.g., ectopic thyroid gland), partial defects in the DUOX2 enzyme complex (*DUOX2*, *DUOXA2*) are the major known genetic risk factor for transient CH^1,2,16–18^. Compared to a cohort of patients with permanent CH, one limited to transient CH cases is likely enriched for *DUOX2* defects. Thus, if the excess risk for IBD in CH patients is due to higher *DUOX2* mutational load, IBD risk is anticipated to be even higher in transient CH (operationally defined as individuals diagnosed with CH not taking levothyroxine (T4) during the observation period) vs permanent CH (defined as those taking T4). Only patients in the CH cohort who had available pharmacy records were included in these analyses. Both permanent and transient CH cohorts had increased prevalence of IBD compared to non-CH controls (Tables 2 and 3), but the odds ratio was significantly higher for transient CH patients (OR 2.39 (95% CI 1.77–3.23) vs 1.69 (95% CI 1.31–2.18), respectively, Tables 4, 5**)**. Compared to permanent CH patients, the prevalence of IBD in the transient CH group was 72% higher (0.81% vs 0.47%; p = 0.0003; non-CH: 0.30%) (Table 6). Similar to the findings for the total CH cohort, the strength of association between transient CH and IBD was highest for indeterminate colitis, followed by UC, and appeared to be weakest for CD.

**Table 2:** The prevalence of IBD in patients with permanent CH (i.e., taking T4) vs. patients without CH (Bivariate comparison).

	Permanent CH N = 24,306	Non-CH N = 42,922	p value
CD	33 (0.14%)	51 (0.12%)	0.5501
UC	47 (0.19%)	54 (0.13%)	0.0298
Indeterminate Colitis	34 (0.14%)	22 (0.05%)	0.0001
Any IBD	114 (0.47%)	127 (0.30%)	0.0003

**Table 3:** The prevalence of IBD in patients with transient CH (i.e., not taking T4) vs. patients without CH (Bivariate comparison).

	Transient CH N = 8,128	Non-CH N = 42,922	p value
CD	21 (0. 26%)	51 (0.12%)	0.0021
UC	28 (0. 34%)	54 (0.13%)	<0.0001
Indeterminate Colitis	17 (0. 21%)	22 (0.05%)	<0.0001
Any IBD	66 (0. 81%)	127 (0.30%)	<0.0001

**Table 4:** The primary endpoint (diagnosis of IBD) in patients with permanent CH vs. non-CH cohort (Multivariable Logistic Regression Models) - summarized models.

Model*	Dependent variable	Odds Ratio**	95% Confidence interval	p-value
1	UC	1.64	1.11–2.43	0.0136
2	Indeterminate Colitis	2.90	1.23–2.38	0.0001
3	Any IBD	1.69	1.31–2.18	<0.0001

*All three models adjusted for age and gender (see Supplemental Table 4 for the complete models).

**The odds of developing an endpoint in patients with CH taking T4 vs non-CH patients.

**Table 5:** The primary endpoint (diagnosis of IBD) in patients with transient CH vs. non-CH cohort (Multivariable Logistic Regression Models) - summarized models.

Model*	Dependent variable	Odds Ratio**	95% Confidence interval	p-value
1	CD	1.77	1.06–2.96	0.0291
2	UC	2.48	1.56–3.94	0.0001
3	Indeterminate Colitis	3.64	1.91–6.91	<0.0001
4	Any IBD	2.39	1.77–3.23	<0.0001

*All four models adjusted for age and gender (see Supplemental Table 5 for the complete models).

**The odds of developing an endpoint in patients with CH not taking T4 vs. non CH patients.

**Table 6:** IBD prevalence in patients with transient CH (not taking T4) vs. permanent CH (taking T4).

	Transient CH N = 8,128	Permanent CH N = 24,306	p value
CD	21 (0.26%)	33 (0.14%)	0.0189
UC	34 (0.28%)	47 (0.19%)	0.0141
Indeterminate Colitis	22 (0.21%)	34 (0.14%)	0.1724
Any IBD	66 (0.81%)	114 (0.47%)	0.0003

## Discussion

In a large cohort of the commercially insured U.S. population, we found an increased prevalence of IBD among individuals with CH compared to those without CH. To our knowledge, this is the first study providing evidence for the existence of a common risk factor for these conditions.

Hypothyroidism, more broadly defined and including common acquired causes, has previously been studied in IBD patients, and its frequency was either found to be unchanged or even lower than in non-IBD controls^19^. Thus hypothyroidism does not appear to be a risk factor in the pathogenesis of IBD. Rather, a suggestive link between IBD and hyperthyroidism, commonly due to autoimmune thyroid disorders, has been found in some studies^20^. This is potentially due to a shared immune pathology, which can lead to increased inflammation of distinct target organs. In light of these prior studies, the clear association between IBD risk and CH in the U.S. population reported here is notable and would indeed support the concept of a shared inborn susceptibility event, such as, variants in the *DUOX2* system that are relevant for thyroid function as well as intestinal immune homeostasis. Interestingly, the association between CH and IBD risk was not related to the severity of CH but was higher for individuals with transient CH. Among the 32,434 CH cohort with available pharmacy claims data, 8,128 (25%) were apparently not treated with T4 and operationally defined as transient CH cases (Tables 2, 3, 6). Although these may include individuals with mild permanent CH in whom treatment had been inappropriately discontinued, the high prevalence of CH cases without ongoing T4 treatment is consistent with an earlier study estimating that by 36 months of age, 38% of US children diagnosed with CH had discontinued T4 treatment^21^. Common genetic defects that frequently manifest as transient-only CH are partial defects in the genes encoding the subunits of the rate-limiting enzyme in thyroid hormone synthesis, *DUOX2* and *DUOXA2*^1,2,16,17^.

*DUOX2* loss-of-function variants appear to be the most common genetic abnormality in CH patients^3–7^. Since DUOX2 is rate-limiting for hormone synthesis, even mild heterozygous defects are anticipated to increase serum TSH level, which varies by one order of magnitude in the general population. However, minor defects are not necessarily sufficient to manifest haploinsufficiency detected by abnormal neonatal CH screening results. In fact, low genetic penetrance for CH is indicated by studies of families in which heterozygous mutation carriers other than the proband were consistently found to have normal thyroid function tests. Furthermore, by reviewing of *DUOX2* variants found in the general population (ExAC database)^22^ we identified a wide spectrum of loss-of-function variants (Supplemental Table 3). While the frequency of individual variants is far below the threshold required for genome-wide association studies, the combined mutational load is substantial. For instance, over 3.2% of the general population harbor one of the previously *in vitro* tested loss-of-function variants (32 variants with *in vitro* activity ≤60% of the reference allele)^23^ with an even higher proportion of individuals carrying uncharacterized, but predicted to be deleterious variants, considerably higher than the prevalence of even mild CH (Supplemental Table 3). Similarly, among East Asians, over 2% carry the Y246X *DUOXA2* complete loss-of-function allele, which has been linked to mild CH in the homozygous state^24^.

Additional risk factors presumably relevant for precipitating disease in carriers of *DUOX2* mutations are distinct for CH and IBD. For CH, the function of the *DUOX1* isoenzyme is likely critical for the potential to compensate partial *DUOX2* defects^25^ and expressivity of the defects is presumably modulated by nutritional iodine intake^1^. Noteworthy, *DUOX1* is expressed in the thyroid epithelial cells but not in the intestine, so compensated partial *DUOX2* defects without associated CH might still have an impact on intestinal immune homeostasis leading to intestinal inflammation. For IBD, a different set of genetic (e.g., *NOD2*) and environmental (e.g., intestinal microbiota) factors are involved in its pathogenesis. It is thus not surprising that among the so-far studied pediatric CH cohorts with *DUOX2* mutations no case of concurrent IBD has been reported, and that the recently described cases of *DUOX2*-associated IBD all had apparently normal thyroid function parameters^13–15^. Based on the high frequency of *DUOX2* mutations in unselected CH cohorts of various ethnic origins^3–7^, our finding of a significantly increased risk of developing IBD among CH individuals lends support for the hypothesis of a shared susceptibility factor, specifically *DUOX2* genetic variants that can affect both thyroid function as well as intestinal immune homeostasis.

An obvious strength of the current study is the large sample size that made testing the main hypothesis feasible. However, several limitations must also be noted. First, there are general limitations of claims based data not collected for research purposes, such as data entry errors and underreporting of diagnoses. To ameliorate the influence of misdiagnoses, we used a 2–3 code requirement in this study. Second, although we matched the study cohorts by age and gender, a generally acceptable method, it is not possible to fully account and correct for other possible confounders (e.g., smoking status, ethnicity).

In summary, we demonstrated a significant association between CH and the development of IBD providing evidence for a shared susceptibility factor. The risk was highest for patients with transient CH, for which partial defects in the DUOX2 system are a frequent genetic cause. Thus our results would seem to support the hypothesis that *DUOX2* mutation carriers, common in the general population, are not only at increased risk for thyroid dysfunction but also impaired intestinal immune homeostasis.

## Methods

### Study Design and Data Source

We performed a retrospective cohort analysis to compare the prevalence of IBD in patients with and without CH. We queried the Truven Health MarketScan databases from January 1^st^, 2009 through December 31^st^, 2015 to identify patients with CH and a matched cohort of patients without CH. The MarketScan databases contain health insurance claims for over 230 million privately insured patients in the U.S. This study was considered exempt by the Institutional review board at the University of Michigan due to the de-identified nature of the dataset.

### Study sample

The International Classification of Diseases, Ninth Revision, Clinical Modification (ICD-9-CM) diagnostic codes were used to identify patients with CH (243.x) and IBD (555.x and 556.x). In order to ensure that CH was confirmed, patients were selected for inclusion if they had at least 2 of the CH ICD-9-CM code in an outpatient setting in 2 different visits while for IBD diagnosis, patients were selected if they had at least 3 ICD-9 codes of IBD in an outpatient setting in 3 different visits between 2009–2015. This approach has a positive predictive value for CD of 0.84 and a positive predictive value for UC of 0.91^26^. We also required at least 1-year enrollment to adequately capture diagnoses and eliminate possible confounding resulting from loss of follow-up. Patients meeting the inclusion criteria for CH were matched in their birth year and sex, in a 1-to-1 fashion, to patients with no CH diagnosis selected from the pool of database participants. If a patient with CH had more than one match, a randomized algorithm was employed to select a match.

### Primary Outcome

The primary endpoint was a diagnosis of IBD in patients with and without CH at any point of the study period. Given the congenital nature of CH, it is safe to assume that IBD manifested after the diagnosis of CH.

### Statistical Analysis

We compared the demographics and prevalence of IBD diagnosis between the two cohorts using the Chi-square test for categorical data, and the Student *t*-test and Mann Whitney U test for continuous data. In sensitivity analyses, we used multivariable logistic regression models to adjust for age and gender in subgroup analyses. Variables (e.g., gender and age groups) were selected for inclusion in the multivariable model if they were significant in bivariate analyses (Supplemental Tables 4 and 5). A two-sided p < 0.05 was used to denote statistical significance. All analyses were performed using Statistical Analysis System (SAS) software version 9.4 (SAS Institute, Inc., Cary, NC). The 1-to-1 matching was performed with R version 3.3.2 (R foundation, Vienna, Austria).

## Electronic supplementary material

Supplementary materials
