# Supplementary material for: Increased risk for inflammatory bowel disease in congenital hypothyroidism supports the existence of a shared susceptibility factor
Source: Sci Rep. 2018 Jul 5;8:10158. doi: 10.1038/s41598-018-28586-5 (PMC6033893; doi:10.1038/s41598-018-28586-5)
Supplement: Supplementary file 1 — Supplementary materials [file 41598_2018_28586_MOESM1_ESM.docx]

384 SUPPLEMENTAL DATA

# **Increased risk for inflammatory bowel disease in congenital hypothyroidism supports the existence of a shared susceptibility factor**

Helmut Grasberger^#1^, Mohamed Noureldin^#1,2^, Timothy D. Kao^#1^, Jeremy Adler^3,4^, Joyce M. Lee^4^, Shrinivas Bishu^1^, Mohamad El-Zaatari^1^, John Y. Kao^1^*, Akbar K. Waljee^1,2^*

^1^Department of Internal Medicine, Division of Gastroenterology, University of Michigan, Ann Arbor, Michigan 48109

^2^Veterans Affairs Center for Clinical Management Research, Ann Arbor, Michigan 48109

^3^Department of Pediatrics and Communicable Diseases, Division of Pediatric Gastroenterology, Michigan Medicine, University of Michigan, Ann Arbor, Michigan 48109

^4^Child Health Evaluation and Research (CHEAR) Center, University of Michigan, Ann Arbor, Michigan 48109

^#^ The authors contributed equally.

*Correspondence:

Akbar K. Waljee, M.D. M.Sc., Associate Professor of Medicine, Division of Gastroenterology, Department of Internal Medicine, Michigan Medicine, Ann Arbor Veterans Affairs Medical Center, 2215 Fuller Road, 111D, Ann Arbor, Michigan 48105

Tel +1 [734 845 5865](mailto:734%20845%205865)

Fax +1 [734 845 3091](mailto:734%20845%203091)

E-mail: [awaljee@med.umich.edu](mailto:awaljee@med.umich.edu)

John Y. Kao, M.D., Associate Professor of Medicine, Division of Gastroenterology, Department of Internal Medicine, Michigan Medicine, 1150 W. Medical Center Drive, SPC 5682, 6520A MSRB 1, Ann Arbor, Michigan 48109

Tel +1 734 647-2964

Fax +1 734 763-2535

E-mail: jykao@umich.edu

386 **Supplemental Table 1.** Demographics of patients with and without CH

| Variables | CH  N = 42,922 | Non-CH  N = 42,922 |
| --- | --- | --- |
| Age, Mean (SD) | 39.1 (25.5) | 39.1 (25.5) |
| Female n (%) | 28,926 (67.4) | 28,926 (67.4) |
| Region Northeast  North Central  South  West  Unknown | 11,923 (27.8%)  8,802 (20.5%)  14,221 (33.1%)  6,827 (15.9%)  1,149 (2.7%) | 7,580 (17.7%)  9,590 (22.3%)  16,041 (37.4%)  8,201 (19.1%)  1,510 (3.5%) |

387

388

1. **Supplemental Table 2.** Prevalence of IBD in patients with CH vs. non-CH (age-and-sex
2. matched cohorts) (3 ICD codes requirement)

392

|  | CH n=37,375 | non-CH n=37,375 | p-value |
| --- | --- | --- | --- |
| CD | 55 (0.15%) | 47 (0.13%) | 0. 4280 |
| UC | 78 (0.21%) | 41 (0.11%) | < 0.0007 |
| Indeterminate Colitis | 62 (0.17%) | 16 (0.04%) | < 0.0001 |
| Any IBD | 195 (0.52%) | 104 (0.28%) | < 0.0001 |

393

394

395

**Supplemental Table 3.** Allele frequency of CH-associated *DUOX2* variants in the general population

| **RSID** | **Consequence** | **Annotation** | **In Vitro Activity (% of wild type)** | **Allele Frequency (%)** |
| --- | --- | --- | --- | --- |
| rs138353181 | p.Ala728Thr | missense | **21** | **0.7866** |
| rs530719719 | p.Phe966SerfsTer29 | frameshift | **15** | **0.2966** |
| rs547116063 | p.Gln570Leu | missense | **25** | **0.1378** |
| rs76343591 | p.Cys1052Tyr | missense | 72/17 | 0.1302 |
| rs113632824 | p.Ala1123Thr | missense | **10** | **0.1281** |
| rs201229193 | p.Glu1546Gly | missense | **55** | **0.0812** |
| rs180671269 | p.Lys530Ter | stop gained | **0** | **0.0635** |
| rs147945181 | p.Leu1343Phe | missense | ND | 0.0553 |
| rs141291775 | p.Arg764Trp | missense | ND | 0.0444 |
| rs8028305 | p.Arg683Leu | missense | ND | 0.0314 |
| rs145061993 | p.Val779Met | missense | ND | 0.0281 |
| rs550037603 | p.Ala1323Thr | missense | ND | 0.0203 |
| rs368488511 | p.Arg1110Gln | missense | **5** | **0.0191** |
| rs766496010 | p.Arg354Trp | missense | **0** | **0.0155** |
| rs200785525 | p.Arg1470Trp | missense | ND | 0.0152 |
| rs144543420 | p.Arg1492His | missense | ND | 0.0148 |
| rs181461079 | p.Arg885Gln | missense | **10** | **0.0108** |
| rs200948626 | p.Met866Arg | missense | **0** | **0.0097** |
| rs368512412 | p.Gly1518Ser | missense | **0** | **0.0080** |
| rs119472029 | p.Arg376Trp | missense | **13** | **0.0079** |
| rs774556391 | p.Glu879Lys | missense | **10** | **0.0076** |
| rs779340990 | p.Gly702del | inframe deletion | ND | 0.0069 |
| rs119472026 | p.Arg434Ter | stop gained | **0** | **0.0069** |
| rs748793969 | p.Ala649Glu | missense | ND | 0.0065 |
| rs200000982 | p.Tyr1150Cys | missense | **19** | **0.0065** |
| rs119472028 | p.Arg842Ter | stop gained | **0** | **0.0051** |
| rs201109959 | p.Arg701Ter | stop gained | **0** | **0.0036** |
| rs747270555 | p.Asn43Tyr | missense | **60** | **0.0033** |
| rs769258094 | p.Gly624AlafsTer15 | frameshift | **0** | **0.0032** |
| rs2467828 | p.Ser199TrpfsTer122 | frameshift | **0** | **0.0028** |
| rs758318135 | p.Leu1160del | inframe deletion | **9** | **0.0025** |
| rs568196384 | p.Trp301Cys | missense | ND | 0.0024 |
| rs147540920 | p.Ala1131Ser | missense | ND | 0.0024 |
| rs752437461 | p.Arg1267Trp | missense | ND | 0.0022 |
| rs200592893 | p.Lys628ArgfsTer11 | frameshift | **0** | **0.0022** |
| rs756822740 | p.Ile1080Thr | missense | **55** | **0.0022** |
| rs769789467 | p.Trp734Ter | stop gained | **0** | **0.0020** |
| rs762588205 | p.Ala1206Thr | missense | **0** | **0.0020** |
| rs774468038 | p.Glu327Ter | stop gained | **0** | **0.0016** |
| rs748194265 | p.Leu1114SerfsTer56 | frameshift | **0** | **0.0016** |
| rs745729386 | p.Ser911Leu | missense | **50** | **0.0016** |
| rs762212416 | p.Pro96Leu | missense | **55** | **0.0012** |
| rs199733766 | p.Ala72Ser | missense | **25** | **0.0012** |
| rs762340753 | p.Tyr1347Cys | missense | 66 | 0.0012 |
| . | p.Arg82Ser | missense | ND | 0.0008 |
| rs769318570 | p.Gln202ArgfsTer93 | frameshift | **0** | **0.0007** |
| rs151261408 | p.Pro303Leu | missense | 75 | 0.0004 |
| rs780614078 | p.Pro341Ser | missense | 61 | 0.0004 |
| rs191759494 | p.Gly488Arg | missense | **1** | **0.0004** |

**Frequency of all CH associated**

**alleles (%) 1.9861**

**Frequency of confirmed loss-of-**

**function alleles [<60% activity] (%) 1.6232**

396

1. **Notes:** Activity of individual variants are taken from a review by Muzza and Fugazzola ^23^. Allele
2. frequency data are from the ExAC database ^22^.

399

400

**Supplemental Table 4.** The primary endpoint (diagnosis of IBD) in patients with CH taking T4 vs. non-CH cohort (Multivariable Logistic Regression Models) – Complete models

| Variables | **UC** |  | **Indeterminate Colitis** | | **Any IBD** |  |
| --- | --- | --- | --- | --- | --- | --- |
|  | Odds ratio (95% CI) | p-value | Odds ratio (95% CI) | p-value | Odds ratio (95% CI) | p-value |
| Male | REF | - | REF | - | REF | - |
| Female | 0.872 (0.559-1.361) | 0.5472 | 0.714(0.401-1.272) | 0.2526 | 0.859(0.644-1.145) | 0.3004 |
| Age 30-45 (yrs) | REF | - | REF | - | REF | - |
| Age 0-15 | 0.054 (0.012-0.236) | <.0001 | 0.041(0.005-0.323) | 0.0025 | 0.031(0.009-0.099) | <0.0001 |
| Age 16-30 | 0.273 (0.099-0.753) | 0.0121 | 0.683(0.261-1.782) | 0.4355 | 0.459(0.271-0.777) | 0.0037 |
| Age 46-60 | 1.013(0.539-1.907) | 0.9670 | 1.168(0.528-2.585) | 0.7012 | 1.053(0.713-1.555) | 0.7954 |
| Age 61-75 | 1.783 (0.979-3.246) | 0.0587 | 0.763(0.310-1.882) | 0.5574 | 1.334(0.903-1.969) | 0.1476 |
| Age 76-90 | 1.494(0.685-3.260) | 0.3132 | 1.959(0.775-4.950) | 0.1551 | 1.356(0.824-2.233) | 0.231 |
| Age > 90 | 2.041(0.589-7.072) | 0.2604 | 0<.01(<0.01->9.9) | 0.9862 | 1.043(0.371-2.927) | 0.9368 |
| No CH | REF | - | REF | - | REF | - |
| CH (permanent) | 1.640 (1.107-2.429) | 0.0136 | 2.904 (1.226-2.383) | 0.0001 | 1.69 (1.31-2.18) | <0.0001 |

**Supplemental Table 5.** The primary endpoint (diagnosis of IBD) in patients with transient CH (i.e., not taking T4) vs. patients with non-CH (Multivariable Logistic Regression Models) – Complete models

| Variables | **CD** |  | **UC** |  | **Indeterminate Colitis** | | **Any IBD** |  |
| --- | --- | --- | --- | --- | --- | --- | --- | --- |
|  | Odds ratio (95% CI) | p-value | Odds ratio (95% CI) | p-value | Odds ratio (95% CI) | p-value | Odds ratio (95% CI) | p-value |
| Male | REF | - | REF | - | REF | - | REF | - |
| Female | 1.015(0.600-1.675) | 0.9550 | 1.011 (0.631-1.621) | 0.9625 | 1.203 (0.594-2.437) | 0.6085 | 1.049 (0.768-1.434) | 0.7635 |
| Age 30-45 (years old) | REF | - | REF | - | REF | - | REF | - |
| Age 0-30 | 0.047(0.011-0.204) | <.0001 | 0.272 (0.115-0.642) | 0.0030 | 0.217 (0.064-0.729) | 0.0135 | 0.159 (0.087-0.291) | <0.0001 |
| Age 46-60 | 0.934 (0.498-1.752) | 0.8324 | 0.834 (0.408-1.704) | 0.6182 | 0.996 (0.412-2.407) | 0.9933 | 0.912(0.601-1.384) | 0.6651 |
| Age 61-75 | 1.148 (0.608-2.167) | 0.6702 | 1.671 (0.863-3.234) | 0.1277 | 0.899 (0.346-2.338) | 0.8275 | 1.281(0.849-1.933) | 0.2372 |
| Age 76-90+ | 1.665 (0.243-1.821) | 0.4271 | 2.391 (1.119-5.109) | 0.0244 | 1.472 (0.479-4.525) | 0.5000 | 1.436(0.856-2.409) | 0.1709 |
| No CH | REF | - | REF | - | REF | - | REF | - |
| CH (transient) | 1.771 (1.060-2.959) | 0.0291 | 2.476 (1.559-3.935) | 0.0001 | 3.636 (1.914-6.906) | <0.0001 | 2.387(1.765-3.229) | <0.0001 |

20
